# Effects of Training and Calibration Data on Surface Electromyogram-Based Recognition for Upper Limb Amputees

**DOI:** 10.3390/s24030920

**Published:** 2024-01-31

**Authors:** Pan Yao, Kaifeng Wang, Weiwei Xia, Yusen Guo, Tiezhu Liu, Mengdi Han, Guangyang Gou, Chunxiu Liu, Ning Xue

**Affiliations:** 1 State Key Laboratory of Transducer Technology, Aerospace Information Research Institute (AIR), Chinese Academy of Sciences, Beijing 100094, China; yaopan19@mails.ucas.ac.cn (P.Y.); guoyusen21@mails.ucas.ac.cn (Y.G.); liutiezhu19@mails.ucas.ac.cn (T.L.); cxliu@mail.ie.ac.cn (C.L.); 2 School of Electronic, Electrical and Communication Engineering, University of Chinese Academy of Sciences (UCAS), Beijing 100049, China; 3 MRC Brain Network Dynamics Unit, University of Oxford, Oxford OX3 9DU, UK; 4 Department of Spinal Surgery, Peking University People’s Hospital, Beijing 100044, China; wangkaifeng@bjmu.edu.cn (K.W.); wx@bjmu.edu.cn (W.X.); 5 Department of Biomedical Engineering, Beijing University, Beijing 100124, China; hmd@pku.edu.cn

**Keywords:** surface electromyogram, cross-session gesture recognition, model calibration, amputee, intelligent prostheses

## Abstract

Surface electromyogram (sEMG)-based gesture recognition has emerged as a promising avenue for developing intelligent prostheses for upper limb amputees. However, the temporal variations in sEMG have rendered recognition models less efficient than anticipated. By using cross-session calibration and increasing the amount of training data, it is possible to reduce these variations. The impact of varying the amount of calibration and training data on gesture recognition performance for amputees is still unknown. To assess these effects, we present four datasets for the evaluation of calibration data and examine the impact of the amount of training data on benchmark performance. Two amputees who had undergone amputations years prior were recruited, and seven sessions of data were collected for analysis from each of them. Ninapro DB6, a publicly available database containing data from ten healthy subjects across ten sessions, was also included in this study. The experimental results show that the calibration data improved the average accuracy by 3.03%, 6.16%, and 9.73% for the two subjects and Ninapro DB6, respectively, compared to the baseline results. Moreover, it was discovered that increasing the number of training sessions was more effective in improving accuracy than increasing the number of trials. Three potential strategies are proposed in light of these findings to enhance cross-session models further. We consider these findings to be of the utmost importance for the commercialization of intelligent prostheses, as they demonstrate the criticality of gathering calibration and cross-session training data, while also offering effective strategies to maximize the utilization of the entire dataset.

## 1. Introduction

Annually, a total of 22,211 amputations of the upper limb are conducted in 27 European Union member states and the United States [1]. Physical disability has a profound impact on the well-being and quality of life of affected individuals, resulting in detrimental physical and psychological consequences [2,3]. Intelligent prostheses have emerged as a prospective technological solution to address this challenge, facilitating fundamental hand movements for amputees [4,5,6,7]. An essential aspect of this innovative approach, referred to as sEMG-based hand gesture recognition, is its ability to decode sEMG from the muscles of the residual upper limbs of amputees as they endeavor to regain control over their hands [8,9].

Nevertheless, the temporal fluctuations observed in sEMG pose a significant challenge to gesture recognition. While pre-trained recognition models demonstrate satisfactory estimation accuracy in intra-session scenarios, their performance tends to deteriorate significantly in cross-session scenarios [10,11]. These deficiencies, which hinder the efficacy of intelligent prostheses from reaching their expected potential [12], can be attributed to various external factors, including but not limited to muscle fatigue, electrode shift, variations of contraction forces, and arm position effects [13]. Thus, pre-trained models with limited training session data are greatly restricted to mitigate these risks [14,15].

Recently, two strategies have been put forth to overcome the existing bottleneck. First, the augmentation of training data sessions demonstrates promise in reducing the temporal variability of sEMG signals. In a study [16], researchers examined inter-day recognition errors by utilizing both fixed and incremental training data. The findings revealed that the use of incremental training data yielded significantly better outcomes in comparison to the use of fixed training data. Second, the utilization of calibration data, derived from the distribution of unseen data, has also demonstrated efficacy in mitigating the impact of EMG variations [11,17,18,19]. It was found that the system with transfer learning (using calibration data) could significantly lessen the impact of electrode shifts (simulating cross-sessions) in the Box and Beans test [19]. However, a limited number of the aforementioned studies utilized data from individuals with amputations or effectively verified the calibration data through multiple experiments conducted across different sessions. The evolution of gesture recognition performance for amputees in the presence of varying quantities of training and calibration data remains uncertain, as does the optimal amount of training data required to achieve an acceptable level of accuracy.

To address the above questions, we conducted a quantitative analysis of sEMG-based gesture recognition using two cross-session databases, including seven sessions that involved two upper limb amputees (from this study) and ten sessions with nine healthy subjects (from a publicly available database). We presented the impact of calibration data, both alone and in conjunction with training data, on performance, as well as the contribution of the number of training sessions and trials to baseline accuracy. The primary contributions of this study can be succinctly outlined as follows: First, we enlisted two chronic amputee volunteers and constructed a sEMG-based gesture database comprising seven sessions, with twenty trials per session for each. Next, we conducted feature visualization using the cross-session databases in order to depict temporal and gesture-dependent feature variation. Then, four datasets were created to evaluate the impact of calibration data on performance. These datasets are named as follows: baseline, calibrated, updated, and cumulative datasets. Finally, we investigated how increasing the number of training sessions and trials affected the pre-trained models in the baseline dataset.

In the subsequent sections of this paper, the Materials and Methods section provides an overview of the data collection, EMG activity detection, and feature engineering and describes techniques for feature variation visualization and approaches for investigating the impact of calibration and training data on gesture recognition. The Results section details the experimental results of the proposed methods. The Discussion section examines the challenges associated with enhancing cross-session performance, evaluates the efficacy of the calibration and training data, and proposes three prospective approaches for addressing cross-session issues in subsequent research. The Conclusion section sums up the main points of this study.

## 2. Materials and Methods

### 2.1. Data Description

Two male amputees were included in this study, one with a 30-year history of right-arm amputation, aged 55 (subject S1), and the other with a 26-year history of double-hand wrist disarticulation, aged 36 (subject S2). Both of them are right-handed and performed experiments with the right hand. Ethical approval was granted by the Institutional Ethics Committees from the Fourth Medical Center of PLA General Hospital, Beijing, China (2020KY038-HS001), and informed consent to participate in the study was obtained from each subject before the experiment. All experiments were performed in accordance with relevant guidelines and regulations.

**Figure 1:**
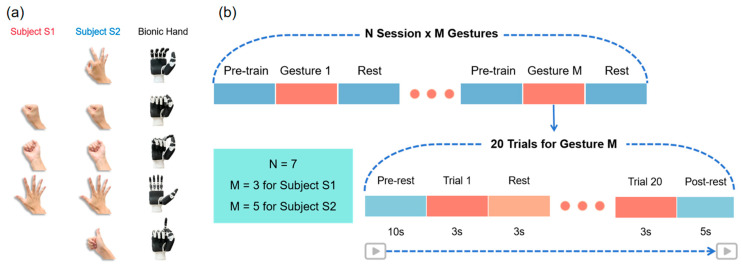
Experiment setup. (**a**) Designed gestures for each subject and their corresponding bionic hand postures. (**b**) The schematic sequence diagram of EMG data acquisition.

First, we designed three daily-use gestures for subject S1 and five for subject S2 (Figure 1a). The difference in the number of gestures performed was largely determined by the subjects’ own reports, related to the degree of amputation and the number of years since amputation. During the initial gesture design phase, we intended for the two participants to perform the same five gestures. However, subject S1 had difficulty performing two of the gestures, so we decided that subject S1 would perform the remaining three gestures. Subject S2, who had a smaller amputation site, had a clear sense of how to perform all five gestures and was therefore asked to complete the full set of gestures. This individualized approach was adopted to cater for individual differences and to ensure that each participant was able to successfully complete the assigned gesture tasks within their respective abilities.

Then, for each subject, the EMG data acquisition was split up into seven independent sessions (Figure 1b). Every session began with a fresh set of electrodes and the same content of collection tasks. Each session involved three gesture acquisitions for subject S1, wherein the three gestures were performed sequentially, and five gesture acquisitions for subject S2, wherein the five gestures were performed sequentially. In each acquisition, every gesture was executed for three seconds with a three-second break in between, for a total of twenty trials of gestures. Seven sessions were completed within 13 days for subject S1 and 4 days for subject S2 (Supplementary Table S1). The minimum time interval between sessions in a day was 1.5 h.

**Figure 2:**
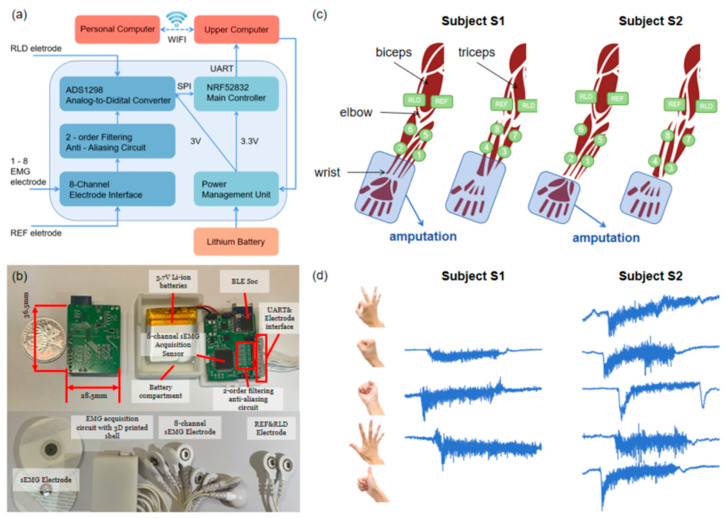
System of sEMG acquisition. (**a**) Block diagram of multi-channel sEMG acquisition circuit. (**b**) Multi-channel sEMG acquisition device. (**c**) Electrode locations for the two subjects. 1–8 represents electrode channels 1–8, RLD represents the right leg drive electrode, and REF represents the reference electrode. (**d**) Raw sEMG signals.

The same EMG recording system, which included a commercial microcomputer and a self-designed signal acquisition circuit (Figure 2a), was used in all experiments. The signal electrode number was designed as eight for the EMG signal acquisition circuit (36.5 mm × 28.5 mm × 4 mm), with the REF electrode serving as the unified reference electrode and the RLD electrode functioning as the input for the right leg driver to reduce common-mode interference. A second-order passive low-pass filter analog front-end circuit was designed to filter out high-frequency noises before signal digitization. A low-power ADS1298R chip was used for physiological electrical signal measurement with an 8-channel 24-bit analog-to-digital converter. As the main control chip, the NRF52832 controls the ADS1298R chip through the SPI communication protocol to collect 8-channel sEMG signals, sending them to the upper computer through a serial port. The upper computer is deployed on a commercial microcomputer, the Cortex-A72 (ARM v8) 64-bit SoC @ 1.5 GHz Raspberry Pi 4B/8GB platform (9.4 cm × 6.9 cm × 2.5 cm), and communicates with a personal computer (Intel (R) Core (TM) i7-8750H CPU @ 2.20 GHz 2.21 GHz) through Wi-Fi for data transmission and command execution. The system is powered and rechargeable using lithium batteries, which have the advantages of small size, portability, and easy use (Figure 2b). The schematic diagram of the EMG signal acquisition circuit is shown in Supplementary Figure S1.

The position of the electrodes for the two subjects is shown in Figure 2c. The REF electrode was placed on the inner side of the upper big arm near the elbow, and the RLD electrode was placed on the outer side of the right upper arm near the elbow. Eight monopolar electrodes were placed on the right forearm. Channels 1–4 were placed uniformly at the anterior portion of the residual limb (from the amputation site to the elbow) and channels 5–8 were placed at the posterior portion of the residual limb. The positions of medical gel electrodes (CH50B, Shanghai Hanjie Electronic Technology Co., Ltd., Shanghai, China) attached to each subject remained almost constant for each data collection session. Figure 2d shows the raw sEMG signals collected through the acquisition circuit from channel 1, depicted for each gesture performed by both subjects.

To broaden the applicability of our experimental results, we also conducted tests on a publicly available database, Ninapro DB6. This database is built to study the repeatability of sEMG signals in gesture recognition [14]. The data are recorded from 10 intact subjects repeating 7 grasps 12 times (trials), twice a day for 5 days (10 sessions in total). Each repetition lasts for 4 s and is followed by 4 s of rest. A total of 14 Delsys Trigno double differential sEMG wireless electrodes were used to record the muscular activity of the forearm. These electrodes were equally spaced around the forearm, with the first eight positioned at the level of the radio humeral joint and the remaining 6 situated below. The electrodes were fixed on the forearm using their standard adhesive bands. Moreover, a hypoallergenic elastic latex free band was placed around the electrodes to keep them fixed during the acquisition. The sEMG signals were sampled at a rate of 2 kHz. Data from subject 9 were removed from this study because the signal from one channel was missing. Therefore, data from a total of nine subjects were investigated in this study.

### 2.2. Signal Preprocessing

Data preprocessing in this study involved EMG signal preprocessing, EMG activity detection, and feature extraction. The signals were divided into frames using a sliding window of 128 ms with a 64 ms overlap, with a sampling frequency of 1000 Hz. A Butterworth fourth-order zero-phase bandpass filter (20–450 Hz) was selected to eliminate noise, including noise that is inherent to electronic devices (ranging from 0 Hz to several thousand Hz), caused by the quasi-random nature of EMG (ranging from 0 Hz to 20 Hz), and motion artifacts (ranging from 0 Hz to 10 Hz) [20]. The Hanning window was applied to reduce spectral leakage by truncating the filtered frames.

**Figure 3:**
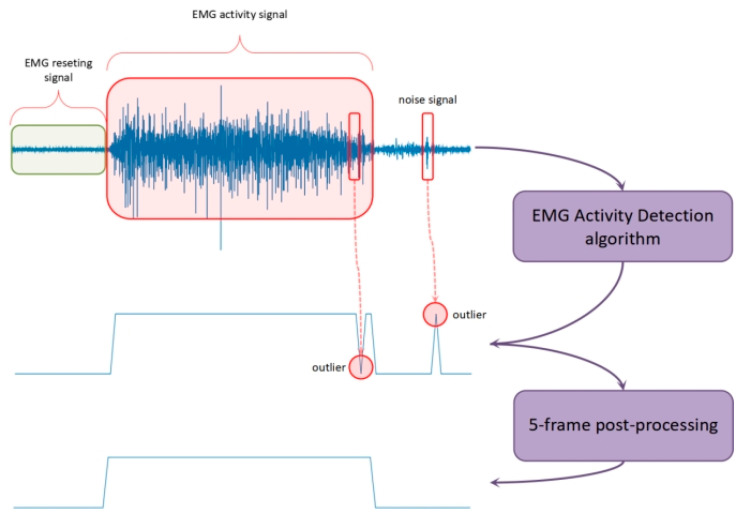
Diagram of EAD.

EMG activity detection (EAD) was then used to determine whether a given EMG signal contains EMG information, i.e., effective EMG corresponding to gesture execution. Typical EAD methods include feature extraction and the determination of EMG/non-EMG signals. In this study, the extracted feature was the short-term energy of sub-bands (Table 1). The decision model was the unsupervised learning method based on the Gaussian mixture model (GMM). The combination of short-term energy of sub-bands and the GMM has been widely used in voice activity detection (VAD) [21]. In this study, we applied this established practice in the realm of EAD. Figure 3 shows the diagram of EAD. First, each pre-processed frame was determined to be either active (1) or resting (0) using the EAD algorithm. However, there would sometimes be misclassifications, as we can see some outliers in the middle of the figure. Therefore, a 5-frame post-processing method was applied as a smoothing function to calibrate the baseline results. We can see that the two outliers were correctly identified and rectified. These frames, with the activity label (1) were used for classification. A total of 14,000 activity frames were extracted for subject S1 and 35,396 activity frames were extracted for subject S2 (Supplementary Table S2) in preparation for the ongoing feature engineering and modeling. We did not perform EAD on Ninapro DB6 because the database was already labelled for active sEMG signals.

Table 1 lists every feature that was extracted for this study, including both intra- and inter-channel features. The intra-channel features are divided two subcategories: time domain features and frequency domain features, containing a total of 14 features for each channel. To capture the short-time energy of sub-band features, the signals were filtered with three different band-pass filters, and then their root mean square values were computed as short-term energy separately, to form a three-dimensional sub-band short-time energy feature vector. For the 4th autoregression coefficient features, four coefficients, as well as the residual, were selected to form a five-dimensional feature vector. The inter-channel feature encompasses Pearson’s correlation coefficient, which has been applied in EEG-based identification [22]. Thus, each active frame associated with an 8-channel signal in our database generates a total of 112 intra-channel features and 28 inter-channel features, resulting in a 140-dimensional feature vector. Similarly, each active frame in Ninapro DB6 produces a 287-dimensional feature vector.

**Table 1:** Feature extraction.

Feature Extraction	Feature Set	Feature Name
Intra-channel (Feature number: 14 × 8 = 112 for this study, 14 × 14 = 196 for Ninapro DB6)	Time Domain	Short-term Energy of Sub-bands (80–150 Hz, 150–300 Hz, 300–450 Hz)
		Root Mean Square
		Waveform Length [23]
		Mean Absolute Value [23]
		Zero Crossings [23]
		Slope Sign Changes [23]
		4th Autoregressive Coefficients [24]
	Frequency Domain	Mean Amplitude Spectrum [25]
Inter-channel (Feature number: 28 for this study, 91 for Ninapro DB6)	Pearson’s Correlation Coefficient	Corr_x_y (x = 1:7; y = (x + 1):8 for this study, x = 1:13; y = (x + 1):14 for this study)

### 2.3. Feature Visualization

To address the issue of hand gesture recognition performance degeneration across sessions, it is essential to first visualize the variation of signals. From the aforementioned feature extraction work, it is possible to generate a graph depicting the evolution of feature distribution over time. Two methods of feature visualization are introduced here, either individually or in combination. First, we utilized a probability density curve for independent feature visualization to observe the evolution of the distribution of a single feature over time. We calculated the kurtosis and skewness of the distribution to further quantify the changes in the distribution. Next, we utilized principal component analysis (PCA) for comprehensive feature information visualization to demonstrate the degree of feature distribution overlap for a single gesture over time.

### 2.4. Cross-Session Validation

**Figure 4:**
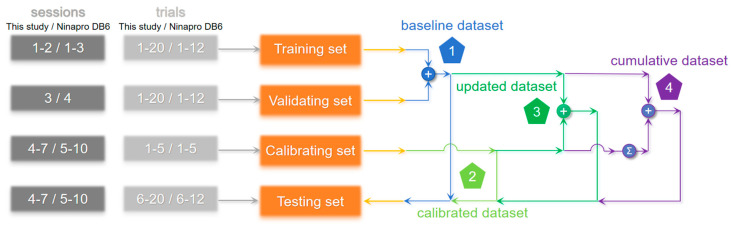
Schematic diagram of cross-session validation for this study and Ninapro DB6.

We aimed to examine the efficacy of gesture recognition in cross-session data and explore strategies for optimizing model training in real-world applications. To this end, we established four distinct datasets to facilitate cross-session validations. These datasets were derived from four separate sets, as depicted in Figure 4. The training set comprised all trials conducted during sessions 1–2 for this study and during sessions 1–3 for Ninapro DB6, the validating set consisted of all trials conducted during session 3 for this study and during session 4 for Ninapro DB6, the calibration set consisted of the initial 5 trials conducted during sessions 4–7 for this study and the initial 5 trials conducted during sessions 5–10 for Ninapro DB6, and the testing set comprised the final 15 trials conducted during sessions 4–7 for this study and the final 7 trials conducted during sessions 5–10 for Ninapro DB6.

The purpose of the validating set was to ascertain the optimal classifier and its corresponding parameters by training on the training set. The calibrating set consisted of a range of first 1 to 5 trials obtained from the testing session for this study and Ninapro DB6. The classifier was tested on the same testing set to obtain all the final results.

The baseline dataset, comprising the training and validating sets, was used to obtain a benchmark result, which is an empirical way to train a gesture recognition model for prostheses [9]. The calibrated dataset, consisting only of the calibrating set, was used to achieve an intra-session result, without the effort of collecting data beforehand. The updated dataset, including training, validating, and calibration sets, was used to investigate the contribution of calibration data in comparison to the benchmark results. The cumulative dataset, including training, validating, and all previous calibrating sets, was used to evaluate the effects of continuously adding the previous calibration data into the dataset. A comparison of these four datasets will provide insight into the effectiveness of using the calibration data.

### 2.5. Amount of Training Data Validation

We also sought to determine the amount of training data required to achieve relatively satisfactory benchmark results. To this end, we trained our model using a variety of training sets and only evaluated it using the data from the last session (session 7 in this study and session 10 in Ninapro DB6). For this study, the number of training sessions varied between 1 and 6, with each session consisting of training trials ranging from 1 to 20. For Ninapro DB6, the number of training sessions varied between 1 and 9, with each session consisting of training trials ranging from 1 to 12. Therefore, 120 and 108 different training sets, each of varying sizes, were generated for this study and Ninapro DB6, respectively. An analysis of these training sets will provide insight into how the quantity of data, as measured by the number of sessions and trials, impacts the performance of gesture recognition for cross-session datasets.

### 2.6. Classifiers and Evaluation Metrics

Commonly used shallow learning methods were chosen as classifiers due to their simplicity, efficiency, and ease of use compared to deep learning methods. Several classifiers were tested on the baseline dataset for this study first, including KNN, SVM, MLP, LGBM, XGBoost, and LDA (Table 2). The relevant parameter settings for the classifiers can be found in Supplementary Table S3. The SVM classifier showed a slightly high accuracy rate (83.04% on average) and less computation time (4 s on average), and was therefore used in the subsequent tests, where its parameters were kept constant. This ensured that, when comparing performance across different datasets (Figure 4), the results were not affected by variations in classifier parameters. It is worth noting that the primary focus of this work was to investigate the impact of the calibration data on cross-session gesture recognition classification, so we did not search for optimal parameters for each classifier, nor did we work on finding the model with the highest accuracy, as these aspects were not the focus of this study.

The training process involved standardizing the training set before model training. The mean and standard deviation of the training set were then used to standardize the testing set. The following are specifications for each dataset scenario (Figure 4): For the baseline dataset, the model was trained on the training set and validating set, and tested on the testing set. For the calibrated dataset, the model was trained on the calibrating set and tested on the testing set. For the updated dataset, the model was trained on the training set, validating set, and calibrating set, while tested on the testing set. For the cumulative dataset, the model was trained on the training set, validating set, and the accumulated calibrating set, while tested on the testing set.

The classification performance was assessed based on its accuracy, calculated as the number of correctly classified samples divided by the total number. To smooth out the classification results, a post-processing technique was applied. This technique groups three consecutive classification results and selects the mode of the results as the final predicted class. The data processing, visualization of features, classification, and evaluation were all carried out using Python 3.6.

## 3. Results

### 3.1. Feature Distribution Visualization

**Figure 5:**
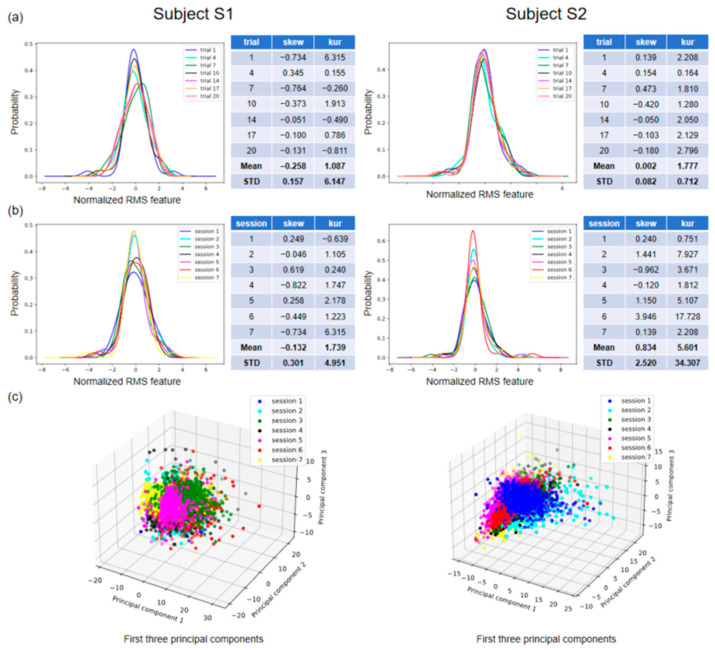
Feature distribution visualization. (**a**) The distribution of the feature RMS of channel 1 for gesture 3 across seven trials in session 7. (**b**) The distribution of the feature RMS of channel 1 for gesture 3 in the first trial across seven sessions. (**c**) The distribution of the first three principal components by PCA for gesture 3 across seven sessions.

Figure 5 shows the distribution of features across varying conditions for the two subjects in our database. Figure 5a,b illustrate the probability density curves for the normalized root mean square (RMS) feature in a single session and across seven sessions, respectively. Specifically, Figure 5a shows the feature distributions for seven trials of gesture 3 in session 7, and Figure 5b shows the feature distributions for the first trial in seven sessions of gesture 3. The skewness (skew) and kurtosis (kur) of each set of distributions, as well as their means (Mean) and standard deviations (STDs), are displayed on the right side of the visualization plot. The standard deviation can indicate fluctuations in the data and, in this context, the degree of variation in the feature distributions. The standard deviations of skew and kur are 0.301 and 4.951 for subject 1, and 2.520 and 34.307 for subject 2 (Figure 5b). By conducting a visual analysis of the distribution of individual features, it becomes evident that there is variation in the distribution not only within a single session but also across multiple sessions.

Figure 5c depicts the tridimensional distribution of the first three principal components across the seven sessions of the identical gesture for each participant. This visualization has the potential to provide a more comprehensive understanding of the variation in distribution across different sessions. This is because principal component analysis (PCA) is capable of capturing a wider range of information and effectively reducing the dimensionality of the data, thereby eliminating noise and irrelevant features while retaining the most significant ones. We can see that the feature distributions of the same gesture under different sessions do not exactly overlap, e.g., session 3 and session 5 for subject 1 and session 1 and session 6 for subject 2 (Figure 5c).

These feature distribution visualizations show, from a data-driven perspective, that achieving generalization for machine learning is challenging in the context of gesture recognition.

### 3.2. Overall Comparison of the Four Datasets

Table 3 displays the overall performance and eventual improvement for different datasets in this study and Ninapro DB6. In the results of this study, the accuracy of the final dataset (cumulative dataset) is improved in nine out of ten instances (except for one calibration trial for S1, which is reduced by 0.16%). As the number of calibration trials for each type of dataset increases, so does accuracy, which is consistent with the expectations. This enhancement differs between individuals and calibration trials. For example, as the number of calibration trials increases from one to five, the accuracy of the calibrated dataset for subject S1 increases by 15.44% (from 71.31% to 86.75%), while that of the cumulative dataset increases by 9.41% (from 75.21% to 84.62%). However, the improvement appears to be less pronounced for subject S2, with a 9.15% improvement for the calibrated dataset and a 2.03% improvement for the cumulative dataset. There is not always an improvement in accuracy when comparing the results longitudinally by fixing the number of calibration trials. For subject S1, the accuracy decreases when the calibration trials number is set to 3, 4, and 5. For subject S2, the accuracy drops at five calibration trials. The results from Ninapro DB6 show an improvement in accuracy within the final dataset (cumulative dataset) across all instances. Similarly, an increase in the number of calibration trials improves accuracy. However, the cumulative dataset does not always outperform the updated dataset either. When the number of calibration trials reaches three, the accuracy of the updated dataset surpasses that of the cumulative dataset.

To provide a macro-level interpretation of the findings, it is observed from the final column (Average) of Table 3 that the inclusion of calibration data has the potential to enhance accuracy to a certain extent. Specifically, the accuracies in this study improve by 6.16% and 3.03% for subject S1 and subject S2, respectively, and by 9.73% for Ninapro DB6. These results validate the effectiveness of calibrating gesture recognition models in intelligent prostheses for everyday applications.

### 3.3. Effect of Increasing the Amount of Training Data on Performance

**Figure 6:**
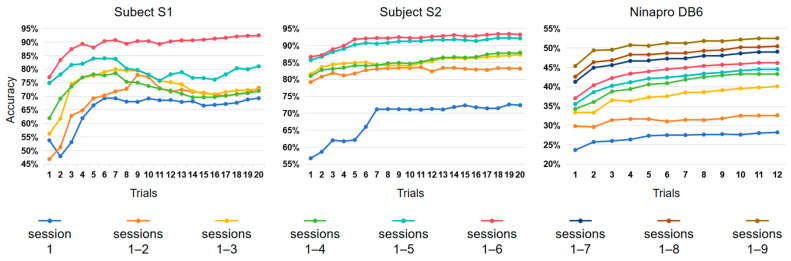
Effects of the amount of training data on gesture recognition.

Figure 6 illustrates how the quantity of training data affects gesture recognition performance. To simplify matters, we limited our experiment to the baseline dataset, without considering the calibration data. As expected, performance improved with an increased number of collected sessions and trials.

However, there exists a discernible pattern of diminishing marginal benefits when the number of trials is increased. Substantial increases in accuracy, followed by fluctuations, are observed during the initial six trials for subject S1 and during the initial seven trials for subject S2. In the case of subject S1, the curve of session 1 exhibits an initial increase from around 55% (one trial) to 70% (seven trials), followed by a period of stability to the end (twenty trials). Regarding subject S2, the progression of the curve sessions 1–6 exhibits an initial increase from 86% (one trial) to 92% (five trials), followed by a period of stability to the end (twenty trials). In the results from Ninapro DB6, the curve of sessions 1–6 shows an initial improvement from 36% (one trial) to 45% (seven trials), followed by a slight increase to just above 45% (twelve trials).

**Figure 7:**
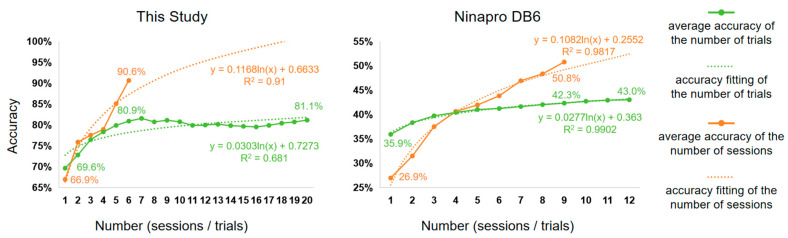
Average accuracy of the number of trials and the number of sessions and the accuracy curve fitting.

To further explore the effect of the number of trials and the number of sessions on gesture recognition, we show their effects on the average accuracy (AA) in Figure 7. The AA was calculated from the results in Figure 6. For example, for this study (the left side of Figure 7), when considering the AA of the number of trials, we averaged the accuracy of six session numbers (one session to six sessions) at each number of trials (one trial to twenty trials). When considering the AA of the number of sessions, we averaged the accuracy from twenty trial numbers (one trial to twenty trials) at each session count (one session to six sessions).

The experimental results suggest that augmenting the number of sessions yields greater efficacy compared to increasing the number of trials. For example, the results from this study show that when increasing the number from one to six, the AA of the number of sessions increases by 23.7% (from 66.9% to 90.6%), while the AA of the number of trials increases by only 11.3% (from 69.6% to 80.9). For Ninapro DB6, when increasing the number from one to nine, the AA of the number of sessions increases by 23.9%, while the AA of the number of trails increases by only 6.4%.

The relationship between AA and the number of trials or sessions is like a logarithmic function. As the amount of data increases, the accuracy should converge towards 100%. Therefore, we fit a logarithmic function to model this relationship, labelling the fitting formula and the goodness-of-fit (R^2^). The fitting curves are consistent with the experimental findings mentioned above. The slope of the fitted curve for the number of sessions is larger than that of the curve for the number of trials. This indicates that the former is more effective in improving accuracy in sEMG-based gesture recognition.

As there are no established criteria for the amount of baseline data required before proceeding with the use of prostheses, these experimental findings highlight the significance of collecting a sufficient number of sessions.

## 4. Discussion

### 4.1. Difficulties in Improving Cross-Session Performance

This study aimed to provide insights into improving gesture recognition performance in real-world scenarios by assessing the quantitative impact of training data and calibration data on cross-session datasets. It is important to acknowledge that the electrode positions for each session were not recorded, and the electrodes were not firmly attached to a fixed position. Instead, each electrode had a general position on the arms. We consider this approach to be more effective in simulating the electrode shifts.

Figure 5 shows that the feature distributions across sessions have a moderate degree of overlap. In the same way [16], histograms of time-domain features were presented at three distinct sensor placements across sessions. It is concluded that alterations in the sEMG data distribution resulted in gesture recognition instability over several days [16]. In addition, it is not possible to ensure absolute stability in the performance of sEMG data collected from the same sensor placement for extended periods of time [16]. Nevertheless, cross-session is not the sole determinant of the fluctuations that occurred in sEMG over time. It is evident from Figure 5a that variations can occur even when the electrodes are fixed during one session.

Bingbin et al. further analyzed the correlation between the stability of the feature distribution and cross-session gesture recognition performance based on sEMG [26]. Forty features in the time domain and the frequency domain were extracted and the stability of feature distributions was quantified using the following metrics: the coefficient of variation of the first four moments (CoV) and two-sample Kolmogorov–Smirnov test statistics (K–S). The selected metrics were found to correlate to some extent with classification accuracy. These results suggest that stable temporal changes may be an acceptable way for selecting robust features in long-term studies. We believe that the stability of the inter-channel feature over time is also worth studying. Furthermore, high-density surface EMG electrodes can be further used to study the effect of electrode position on the stability of sEMG feature distribution [27].

Given that sEMG signals inherently vary both within and between sessions, it is worthwhile to explore methods for reducing the variation in feature distribution from both feature engineering and machine learning perspectives.

### 4.2. Effectiveness of Calibration Data

We first demonstrated that incorporating calibration data can mitigate sEMG variations. To illustrate, Table 3 depicts how calibration data influence the gesture recognition model. Experimental evidence suggests that the inclusion of calibration data in baseline datasets (updated and cumulative datasets) leads to improved performance. Furthermore, performance tends to increase as the amount of calibration data increases. One potential rationale could be that the calibration data are generated from a partial distribution of the testing data, wherein the physiological characteristics of the subjects remain consistent, and the positions of the electrodes are held constant. Additional calibration data collected for the updated dataset tend to increase its similarity to the testing dataset, thereby mitigating the effects of electrode shifts and physiology-induced variation.

However, the cumulative dataset does not provide expected benefits over the updated dataset, despite containing more calibration data. Table 3 (last column) shows that our database results indicate only a slight improvement in the cumulative dataset compared to the updated dataset. The performance of the cumulative dataset from Ninapro DB6 is even lower than that of the updated dataset. Therefore, further evaluation of the performance of the cumulative dataset is necessary.

### 4.3. The Effectiveness of Training Data

We also demonstrated that augmenting the amount of training data can reduce the risks of sEMG variations. Expanding the training data has a profound effect on gesture recognition, as illustrated in Figure 6. For subject S1, sessions 1–6, with 20 trials (around 93%), result in a 23% increase in accuracy compared to session 1, with 20 trials (around 70%).

Although an increase in both the number of sessions and trials contributes to performance improvement, their contributions are different. Our database and Ninapro DB6 show a consistent relationship between the number of sessions and trials and the average accuracy (Figure 7). It is clear that increasing the number of sessions is more effective than increasing the number of trials for improving accuracy.

The distribution of data collected in a single session tends to be similar, which is why the marginal benefit decreases as the number of trials increases. Figure 7 (left) shows that the first seven trials in our database make the most significant contribution to the accuracy of gesture recognition, remaining at about 81% thereafter. The first four trials in Ninapro DB6 make significant contributions, with some contributions from the subsequent six trials. However, due to the limited number of trials in this dataset, it is challenging to determine the point at which the accuracy rate stabilizes. Increasing the number of sessions is more efficient because multiple sessions account for different electrode shifts, the subject’s varying physiological states, and other factors. Consequently, the test data distribution is more likely to overlap with that of previous sessions.

The aim of these experiments is to determine the optimal amount of training data required to achieve acceptable accuracy in gesture recognition. We suggest that approximately 10 trials are sufficient for a single session to achieve good accuracy. Further increasing the number of trials may not result in a significant improvement in accuracy. The current experimental results do not provide an optimal outcome in terms of the number of sessions. This is due to the absence of an inflection point, indicating that the current number of sessions is enough.

Additionally, we hypothesize that the optimal training data should be relevant to the number of gestures recognized. The more categories that require recognition, the more challenging the recognition process becomes, and the more training data is necessary to optimize the classifier. This hypothesis could be supported by our experimental results, as our database only contains three (subject S1) and five (subject S2) types of gestures to be identified, whereas Ninapro DB6 contains seven types of gestures to be identified. In Figure 7, after six sessions, our database achieved an accuracy of 90.6%, while Ninapro DB6 only reached around 42%. This allows for investigating the influence of the number of gesture categories on the optimal amount of training data, based on Ninapro DB6 and other publicly available databases.

We believe that our findings could aid in the commercialization of intelligent prostheses, benefiting their users. Intelligent prosthetic systems require personalized gestures and user-specific training data for accurate gesture recognition. When collecting data, one needs to consider the number of sessions and trials, as they can affect long-term accuracy. It is also advisable for users to calibrate the system before use to ensure stable performance. The user has discretion over the amount of calibration data collected before use. As usage time increases, calibration data accumulate, enriching the distribution of training data and improving system performance. It is important to note that the system can still operate satisfactorily even without calibration data collection, as it may include calibration data from multiple sessions within the existing training data.

### 4.4. Future Work

In light of our findings, three approaches that have been previously tested may be investigated to further improve the performance of long-term gesture recognition models for amputees in this study.

First, deep learning-based models could be used for sEMG-based cross-session gesture recognition. Since this study aimed to investigate the impact of calibration data and training data on cross-session gesture recognition, we selected only simple machine learning algorithms for modeling to quickly achieve baseline results and enable the rapid evaluation of the proposed methods. Relevant studies, however, have shown that deep learning can improve gesture recognition across sessions [28,29,30]. Stacked sparse autoencoders (SSAEs) were evaluated for the classification of sEMG and intramuscular recordings in ten able-bodied individuals and six amputee subjects. In the between-day analysis, SSAEs outperformed LDA [30]. An efficient concatenated convolutional neural network (E2CNN) was proposed for classifying sEMG, providing a good response time, with a high-performance accuracy of 98.31 ± 0.5% and 97.97 ± 1.41% for both nondisabled and amputee subjects [29]. Hence, it is advantageous to implement deep learning on the datasets utilized in this work.

Second, calibration data could be utilized to fine-tune the pre-trained models via transfer learning. Although calibration data are also used in this work, the proposed datasets are not part of transfer learning. Transfer learning, which frequently employs deep learning algorithms, shows potential in addressing domain shift issues [16]. These issues frequently arise in cross-session gesture recognition scenarios and occur when a model trained in one domain (sessions to collect training data) cannot be adapted to another domain (sessions to collect testing data). A deep learning-based architecture to calibrate a pre-trained gesture recognition model was developed in a study, where it was discovered that recalibrating with a small quantity of single-session data (referred to as calibration data) resulted in an accuracy of 79.5% for that session [31]. This is in contrast to architectures that either learn exclusively from the training data or solely from the single session (55.2% and 49.6%, respectively). In another study [10], a novel multi-task dual-stream supervised domain adaptation (MDSDA) network, based on a convolutional neural network (CNN), was proposed, which outperformed both CNN and fine-tuning across four train-test estimations. Therefore, these transfer learning methods can re-evaluate the validity of the updated and cumulative datasets proposed in this study.

Third, the models could be routinely fine-tuned through the use of self-recalibration. Fine-tuning the model routinely is attainable by assessing the prediction outcomes of unseen data (which will be the self-recalibration data) that have been primarily corrected via a multi-vote method [32]. It is assumed that adjacent sEMG segments most probably correspond to the same category of hand gestures. In a study, a self-recalibration system demonstrated an average improvement in classification accuracy of <2.99% (11 amputees, 10 movement types) over five testing trials when using self-calibration data as compared to the unrecalibrated classifier [32]. Utilizing long-term daily data, this self-calibration technique holds promise for enhancing the training data and bolstering the robustness of the prosthetic system without requiring additional effort to calibrate the system.

## 5. Conclusions

In order to mitigate the decline in gesture recognition performance across multiple sessions in the context of intelligent prostheses, we assessed the effectiveness of calibration data and examined the impact of varying amounts of training data on pre-trained models. First, we recruited two subjects who had undergone amputations several years before and collected a total of seven sessions of data from each participant. We also used Ninapro DB6, a publicly available database containing data from ten healthy subjects across ten sessions. These databases provide a valuable opportunity to examine the efficacy of cross-session gesture recognition. Next, by means of feature distribution visualization, it was determined that the variations in sEMG occur not only across different sessions but also within individual sessions. We suggest exploring more methods to reduce the variation in feature distribution. Then, through the comparative analysis of four datasets, the experimental findings demonstrate that the inclusion of calibration data yielded average accuracy improvements of 6.16%, 3.03%, and 9.73% for the two subjects and Ninapro DB6, respectively, when compared to the baseline results. The cumulative dataset is worth further study. In order to enhance the efficacy of training data collection, we conducted a comprehensive analysis of the impact of training data at both the session and trial levels. It was found that increasing the number of single tests has a diminishing marginal benefit in terms of improving accuracy. However, increasing the number of sessions has a more significant effect. We believe that more sessions and trials are needed to determine the optimal amount of data collection. Finally, three potential strategies are suggested for enhancing the cross-session performance of gesture recognition in this research. The significance of these findings lies in their potential to enhance the usability and durability of commercially available intelligent prosthetic devices, thereby leading to an overall enhancement in the well-being and quality of life for individuals with limb loss.

## Figures and Tables

**Table 2:** Performance accuracy and time cost among different models on the baseline dataset.

Models	Subject S1		Subject S2		Average	
	Accuracy	Time Cost	Accuracy	Time Cost	Accuracy	Time Cost
KNN	65.35%	0 s	85.31%	0 s	75.33%	0 s
MLP	72.51%	9 s	90.94%	30.5 s	81.73%	19.75 s
LGBM	66.91%	3 s	89.43%	7.5 s	78.17%	5.25 s
XGBoost	66.48%	7 s	89.10%	25.75 s	77.79%	16.375 s
LDA	75.66%	0 s	89.20%	0 s	82.43%	0 s
**SVM**	**75.37%**	**2 s**	**90.71%**	**6 s**	**83.04%**	**4 s**

**Table 3:** Overall performance and eventual improvement across different datasets.

Database	Dataset	Calibration Trials					Average
		1	2	3	4	5	
Subject 1 (This study)	Baseline	**75.37%**					
	Calibrated	71.31%	79.91%	84.18%	86.37%	86.75%	**81.70%**
	Updated	73.87%	80.94%	82.45%	84.01%	84.99%	**81.25%**
	Cumulative	75.21%	81.67%	82.62%	83.52%	84.62%	**81.53%**
	**Improvement**	**−0.16%**	**+6.30%**	**+7.25%**	**+8.15%**	**+9.25%**	**+6.16%**
Subject 2 (This study)	Baseline	**90.71%**					
	Calibrated	84.41%	87.53%	89.36%	92.10%	93.56%	**89.40%**
	Updated	92.35%	92.95%	93.39%	94.10%	94.88%	**93.53%**
	Cumulative	92.79%	93.26%	93.58%	94.25%	94.82%	**93.74%**
	**Improvement**	**+2.08%**	**+2.55%**	**+2.87%**	**+3.54%**	**+4.11%**	**+3.03%**
Ninapro DB6	Baseline	**50.69%**					
	Calibrated	55.19%	66.13%	70.52%	74.86%	76.82%	**68.70%**
	Updated	56.27%	58.84%	60.89%	62.60%	64.19%	**60.56%**
	Cumulative	56.42%	59.02%	60.72%	62.37%	63.57%	**60.42%**
	**Improvement**	**+5.73%**	**8.33%**	**10.03%**	**11.68%**	**12.88%**	**9.73%**

## Data Availability

The datasets used and analyzed in this study are available from the link https://github.com/PanPanGG/gesture-recognition (accessed on 6 January 2024).

## Supplementary Materials

The following supporting information can be downloaded at: https://www.mdpi.com/article/10.3390/s24030920/s1, Supplementary Figure S1: Schematic diagram of the EMG signal acquisition circuit; Supplementary Table S1: Collected Time of Each Session for Each Subject; Supplementary Table S2: The Number of Activity Frames in Each Session for Each Subject; Supplementary Table S3: Parameter Setting for Different Classifiers Using Scikit-Learn Library in Python.
