# Supplementary material for: Effects of Training and Calibration Data on Surface Electromyogram-Based Recognition for Upper Limb Amputees
Source: Sensors (Basel). 2024 Jan 31;24(3):920. doi: 10.3390/s24030920 (PMC10857392; doi:10.3390/s24030920)
Supplement: Supplementary file 1 [file sensors-24-00920-s001.zip › sensors-2771198-supplementary.pdf]

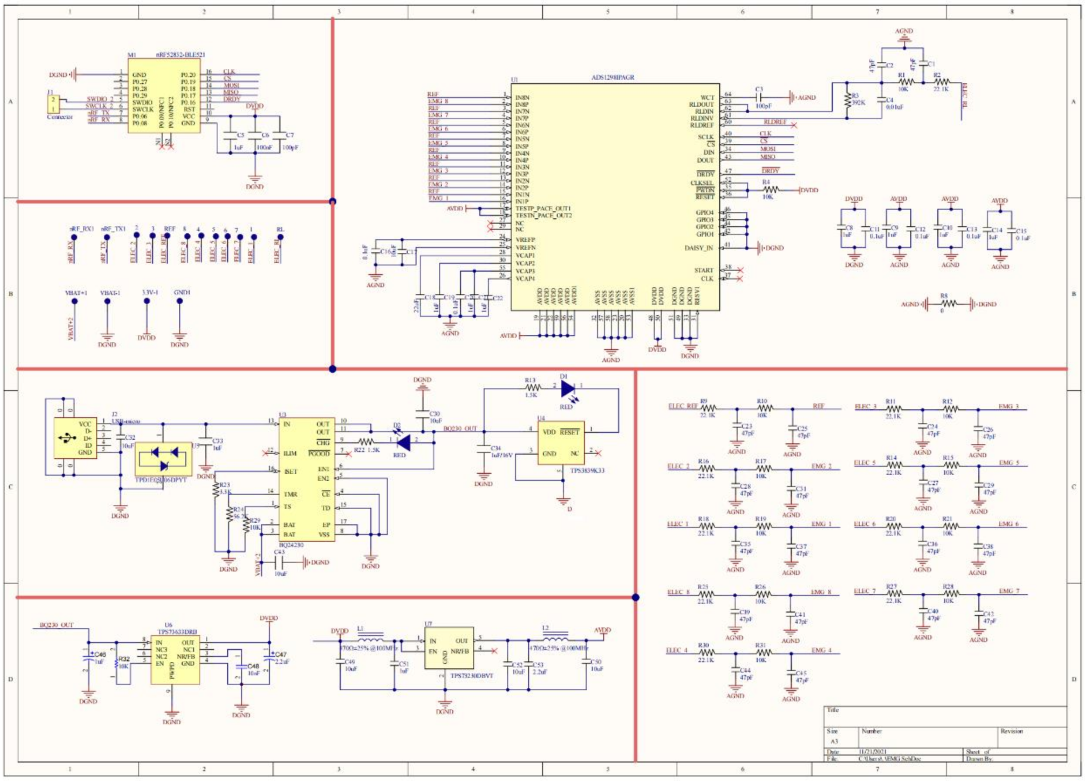

Figure S1. Schematic diagram of the EMG signal acquisition circuit.

Table S1. Collected Time of Each Session for Each Subject.

Session	Time	
	Subject S1	Subject S2
Session 1	Day 1	Day 1
Session 2	Day 1	Day 1
Session 3	Day 1	Day 3
Session 4	Day 3	Day 3
Session 5	Day 13	Day 3
Session 6	Day 13	Day 3
Session 7	Day 13	Day 4

Table S2. The Number of Activity Frames in Each Session for Each Subject

Session	Subject S1	Subject S2
Session 1	2033	5318
Session 2	2379	5029
Session 3	2127	5047
Session 4	2059	5056
Session 5	1842	5484
Session 6	1723	4910
Session 7	1837	4552
In total	14000	35396

TableS 3. Parameter Setting for Different Classifiers Using Scikit-Learn Library in Python.

**Models**	**Function Name**	**Parameters**
KNN	KNeighborsClassifier()	n_neighbors=10
MLP	MLPClassifier()	solver='lbfgs', alpha=1e-5<br>hidden_layer_sizes=(200, 100, 100), random_state=1
LGBM	LGBMClassifier()	Default
XGBoost	XGBClassifier()	max_depth=6, learning_rate=0.05, n_estimators=2000,<br>objective='binary:logitraw',<br>tree_method='gpu_hist', subsample=0.8,<br>colsample_bytree=0.8, min_child_samples=3,<br>eval_metric='auc', reg_lambda=0.5
LDA	LDA()	Default
SVM	SVC()	decision_function_shape='ovr', C=5, kernel='rbf'
